# Supplementary material for: Back to full interseismic plate locking decades after the giant 1960 Chile earthquake
Source: Nat Commun. 2018 Aug 30;9:3527. doi: 10.1038/s41467-018-05989-6 (PMC6117256; doi:10.1038/s41467-018-05989-6)
Supplement: Supplementary file 1 — Supplementary Information [file 41467_2018_5989_MOESM1_ESM.pdf]

Supplementary Materials

Back to full interseismic plate locking decades after the giant 1960 Chile earthquake

Daniel Melnick, Shaoyang Li, Marcos Moreno, Marco Cisternas, Julius Jara-Muñoz, Robert Wesson, Alan Nelson, Juan Carlos Báez, Zhiguo Deng

This file includes Supplementary Tables 1 and 2, and Supplementary Figures 1-13.

Supplementary Table 1. Remote sensing imagery used to estimate relative sea-level changes. These images include material copyright of ©2006 DigitalGlobe, ©2008 IGM, ©2008 SAF, Inc., All Rights Reserved, used with permission under a NERC-BAS educational license and not included in the Creative Commons license for the article.

Agency	Mission	Year	Catalogue Id	Resolution (m)	Number of GCP
IGM	Trimetrogon	1944	408R3 (Oblique)	-	-
IGM	USAF	1974.95	4087	1.1	23
SAF	CH60	1980.20	7434	0.9	24
SAF	Geotec	1998.18	10928	1.1	22
DG	Quickbird	2005.87	1010010004A2A201	0.6	26
DG	GEO1	2014.60	10504100100D2900	0.5	19

IGM-www.igm.cl; SAF-www.saf.cl; DG-www.digitalglobe.com; GCP-Ground Control Point

Supplementary Table 2. Constrained Viscosity Structure.

GPS Station	GUAF	CSTR	MELK	PMO1	JUTA	ESQU
Distance (km)	60	139	150	204	275	347
Averaged viscosity (Pa s)	8×10^{17}	3×10^{19}	5×10^{19}	6×10^{19}	7×10^{19}	4×10^{20}

** Computed 60 years after the earthquake.

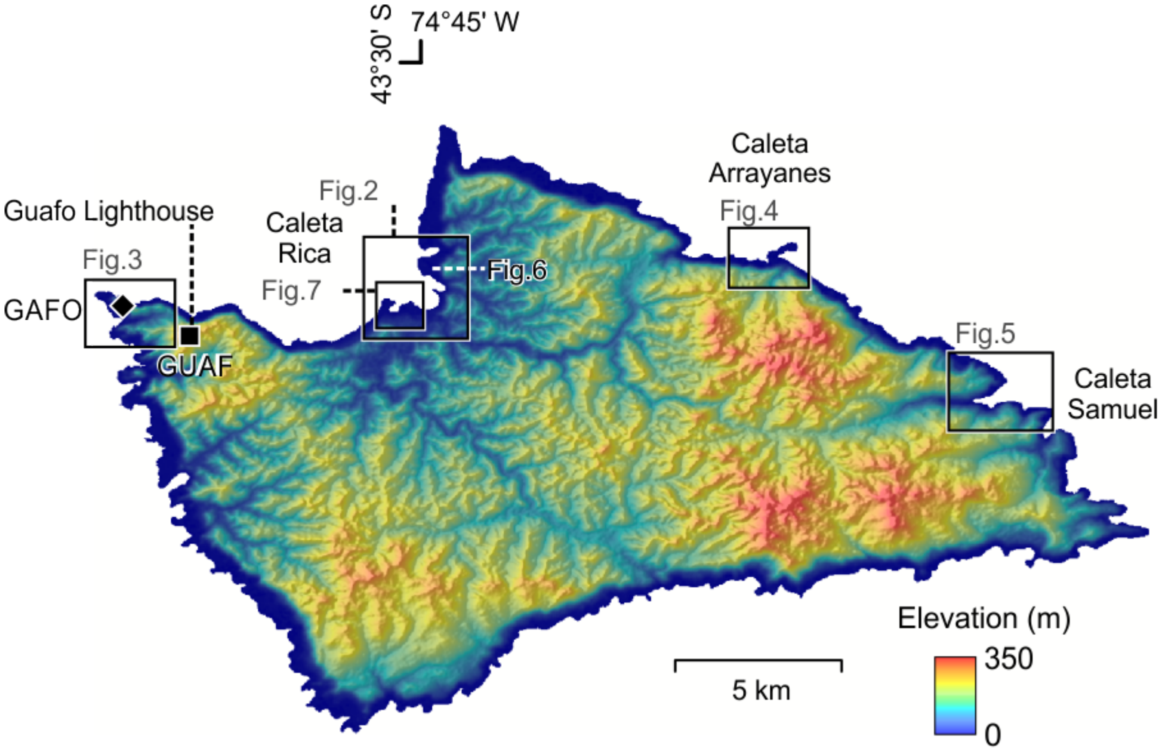

Supplementary Figure 1. Topography of Isla Guafo from NASA Shuttle Radar Topography Mission (SRTM) data[1]. Data available from the U.S. Geological Survey (https://lta.cr.usgs.gov/SRTM). Location of figures in the supplementary materials is indicated. Black diamond and black square show location of the campaign GPS benchmark GAFO and the continuous station GUAF, respectively.

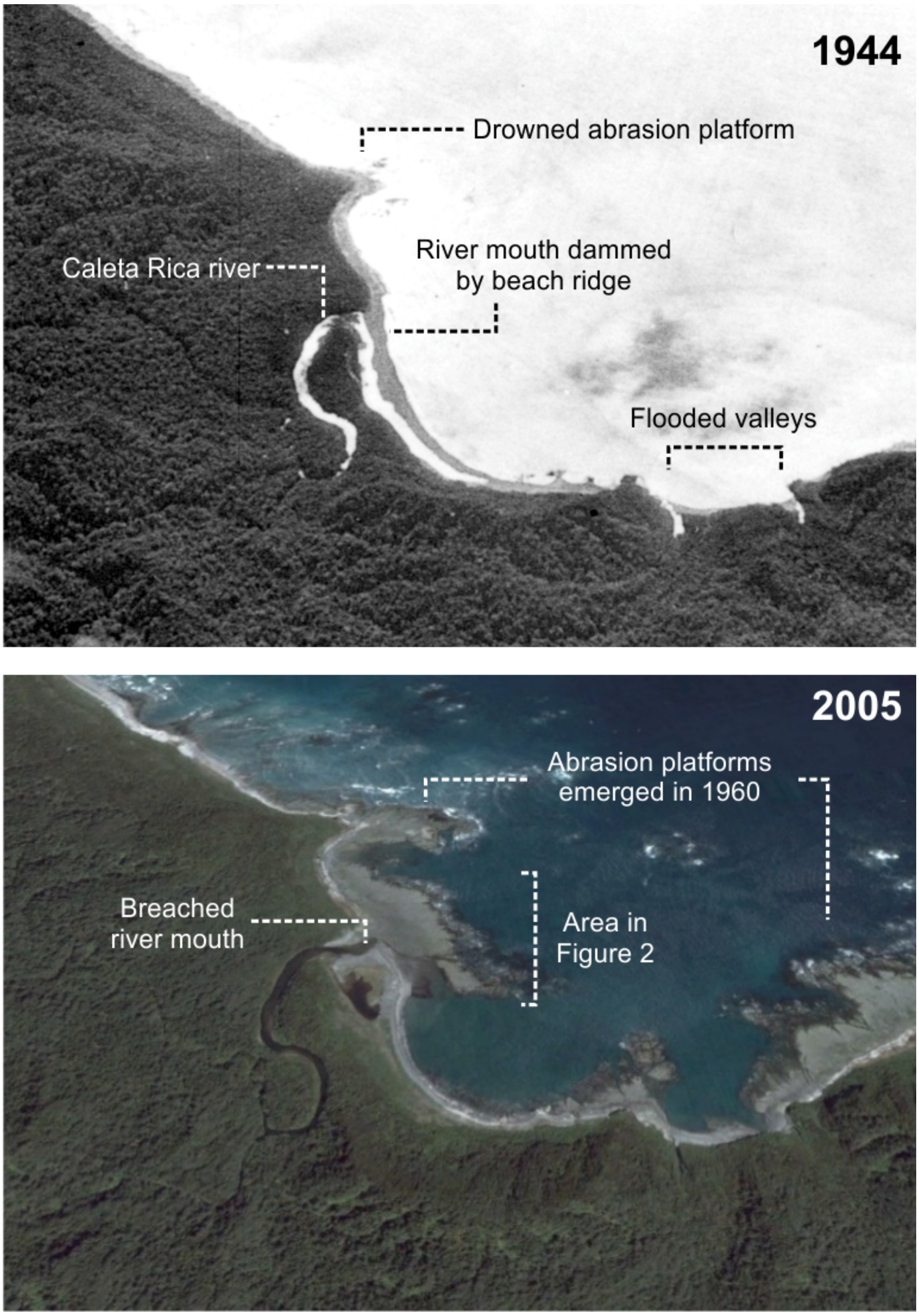

Supplementary Figure 2. Oblique aerial photo from the 1944 Trimetrogon mission (©2008 IGM, Supplementary Table 1) and 2005 Quickbird image (©2006 DigitalGlobe) showing changes in the coastline associated with coseismic uplift in 1960. Note emergence of wide abrasion platforms shown in Supplementary Fig. 7. These images include material copyright of IGM and DigitalGlobe, Inc., All Rights Reserved, used with permission under a NERC-BAS educational license and not included in the Creative Commons license for the article.

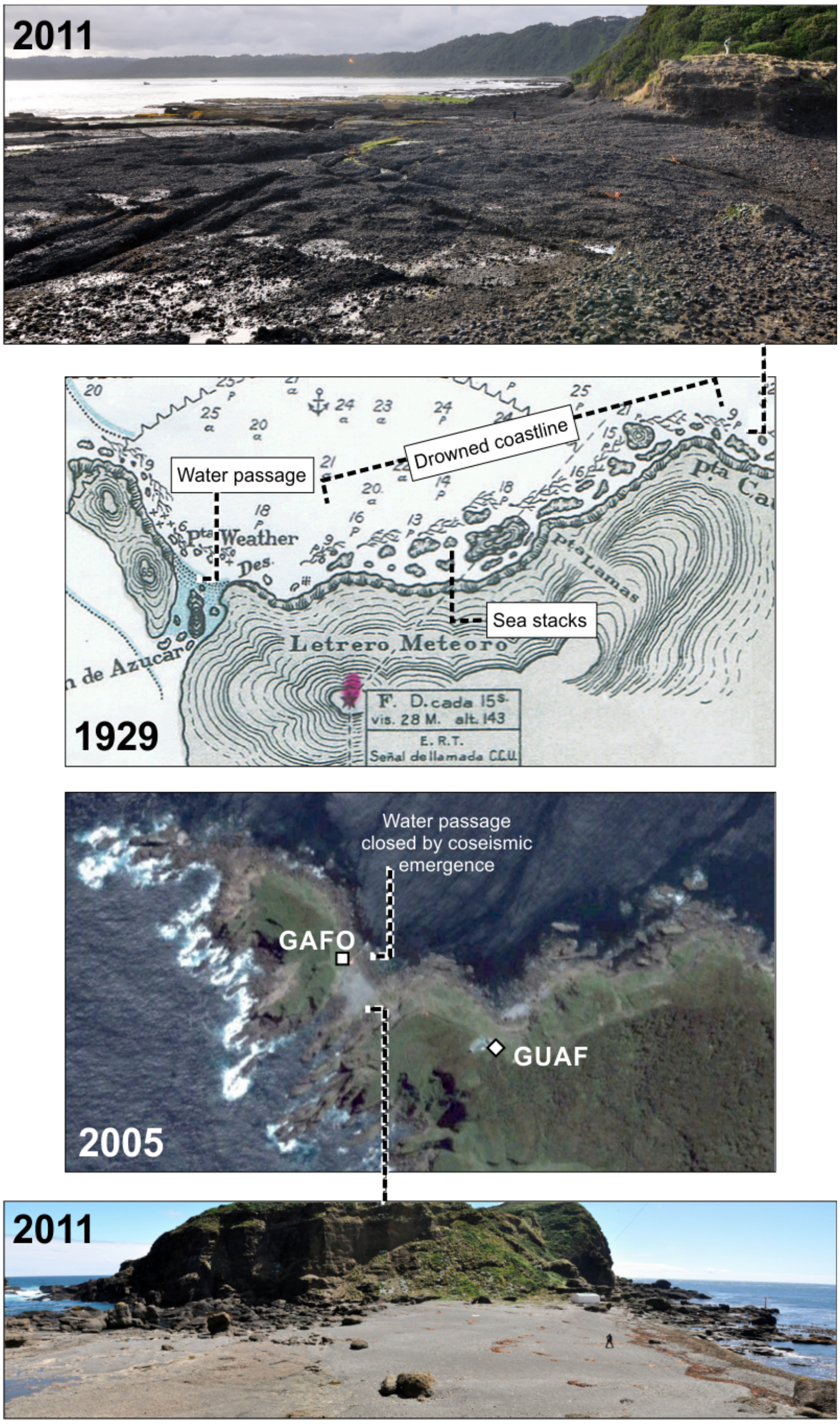

Supplementary Figure 3. Evidence for meter-scale coseismic uplift in 1960 as shown by the closing of a sea passage. Nautical chart from 1929 by the Chilean Navy (©2008 SHOA - Chilean Hydrographic and Oceanographic Survey www.shoa.cl) depicting an open water passage at the northwestern tip of Guafo Island. The same area can be seen covered by beach sand and gravel in a 2005 Quickbird satellite image (©2006 DigitalGlobe, Supplementary Table 1) and a 2011 field view. The gravels covering the passage likely accumulated since coseismic coastal uplift in 1960. The locations of the campaign GPS point GAFO (installed in 1994 by the CAP project) and of the continuous GPS site GUAF installed in 2009 at the Navy lighthouse are labeled on the 2005 image. Field photos by D. Melnick. These images include material copyright of SHOA and DigitalGlobe, Inc., All Rights Reserved, used with permission under a NERC-BAS educational license and not included in the Creative Commons license for the article.

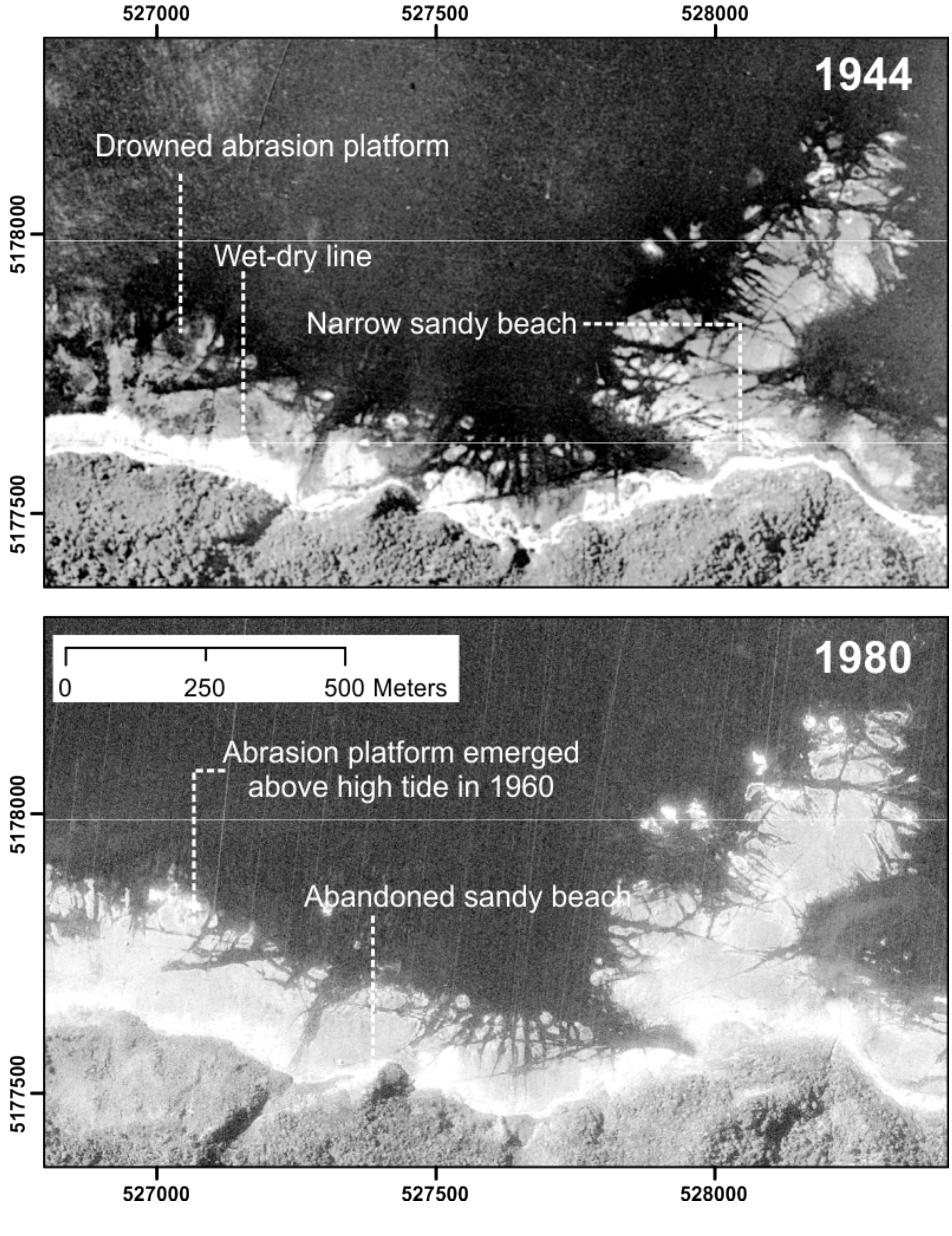

Supplementary Figure 4. Vertical air photos from 1944 and 1980 (©2008 IGM, SAF, Supplementary Table 1) showing geomorphic features associated with coseismic uplift during the 1960 earthquake at Caleta Arrayanes. Note differences in width of sandy beach. These images include material copyright of SAF and IGM, Inc., All Rights Reserved, used with permission under a NERC-BAS educational license and not included in the Creative Commons license for the article.

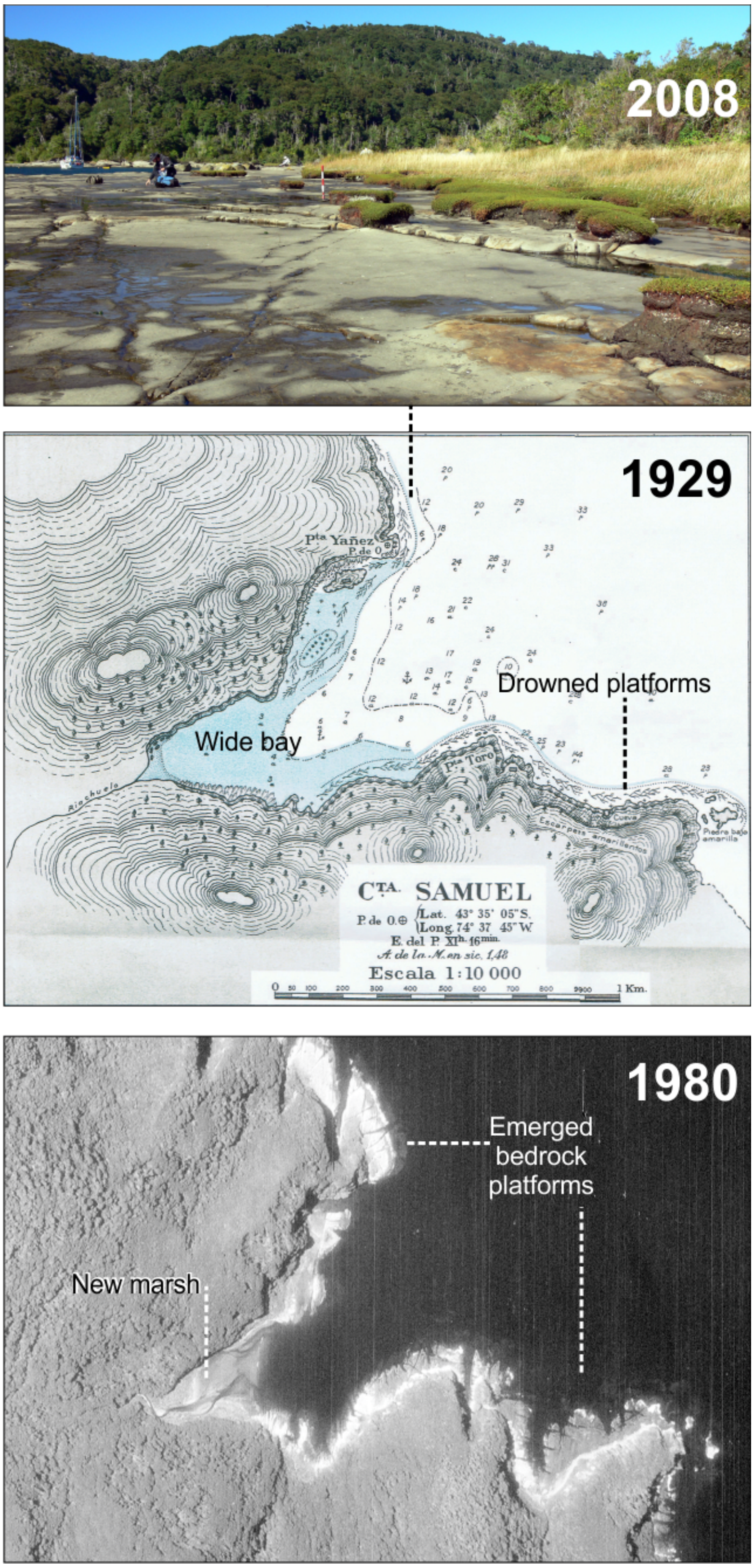

Supplementary Figure 5. Nautical chart from 1929 (©2008 SHOA, www.shoa.cl) and air photo from 1980 (©2008 SAF, Supplementary Table 1) showing geomorphic features associated with coseismic uplift in 1960. Field view from 2008 shows the bedrock platform at Caleta Samuel that was uplifted in 1960 with remains of the post-1960 soil eroded by tides. Field photo by D. Melnick. These images include material copyright of SHOA and SAF, Inc., All Rights Reserved, used with permission under a NERC-BAS educational license and not included in the Creative Commons license for the article.

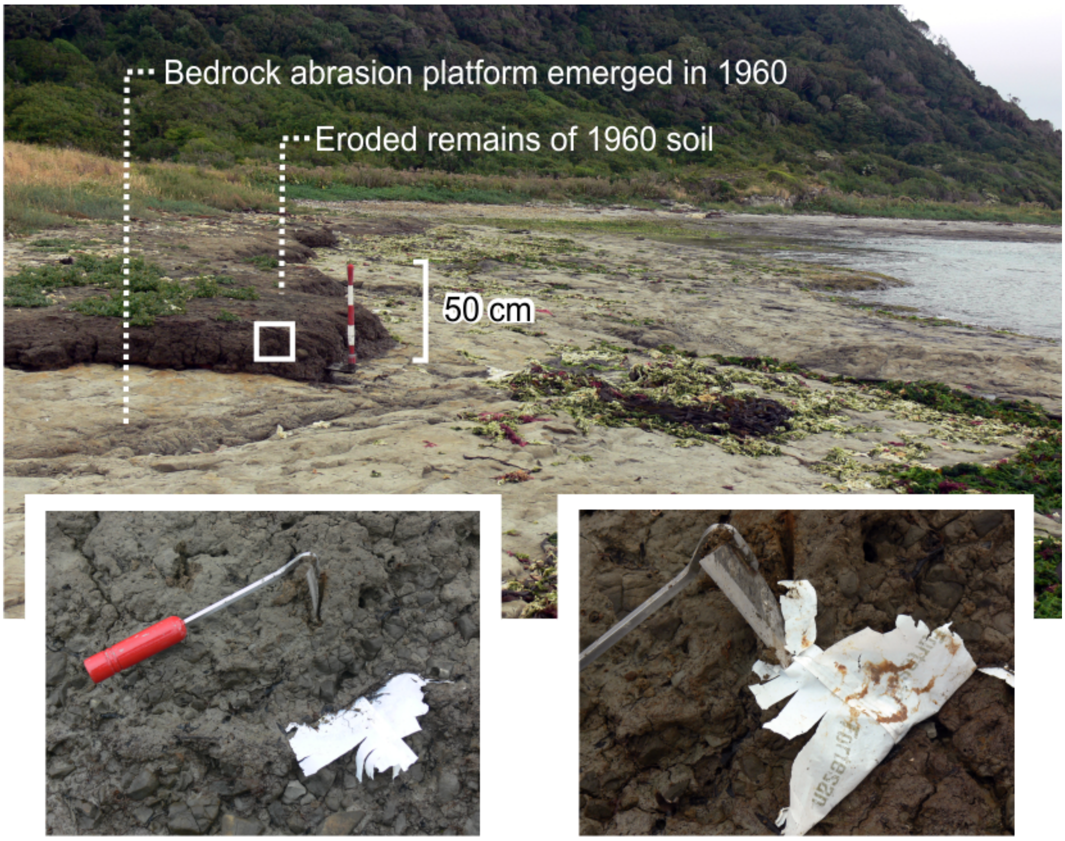

Supplementary Figure 6. Inset shows remains of a package of Fortesan (a soya-based nutritional complement distributed only during the first years of Chile's military government in the mid 1970s) embedded in the soil, which provides a post-1960 age for soil formation. See text for details. Location in Supplementary Fig. 1. Field photos by D. Melnick.

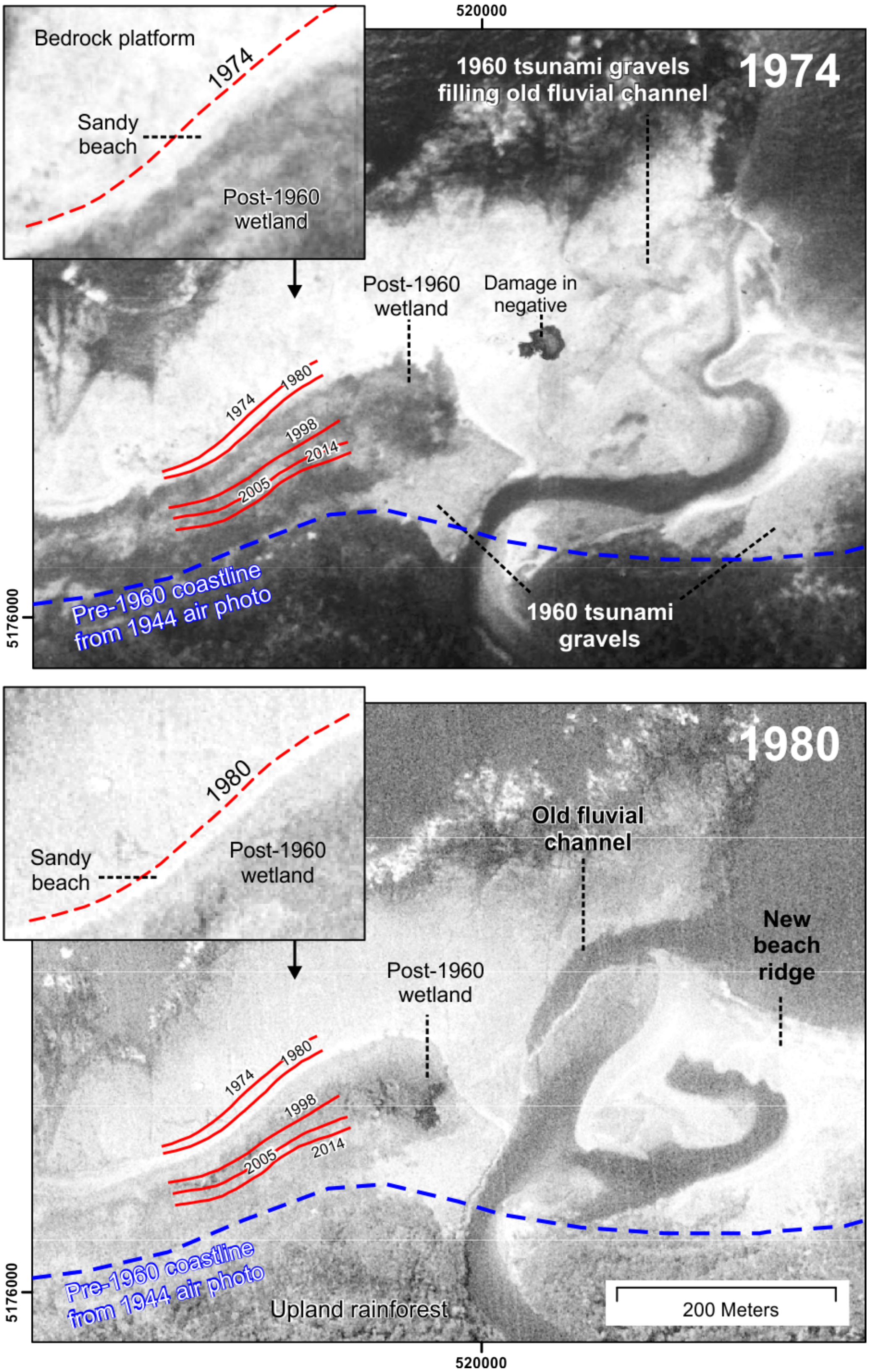

Supplementary Figure 7a. Vertical air photos from 1974 and 1980 (©2008 IGM, SAF, Supplementary Table 1) showing the main geomorphic features and interpreted beach-bedrock platform limits used to estimate relative sea-level changes from historical air photos. Pre-1960 shoreline inferred from 1944 air photo (Supplementary Fig. 2). These images include material copyright of IGM and SAF, Inc., All Rights Reserved, used with permission under a NERC-BAS educational license and not included in the Creative Commons license for the article.

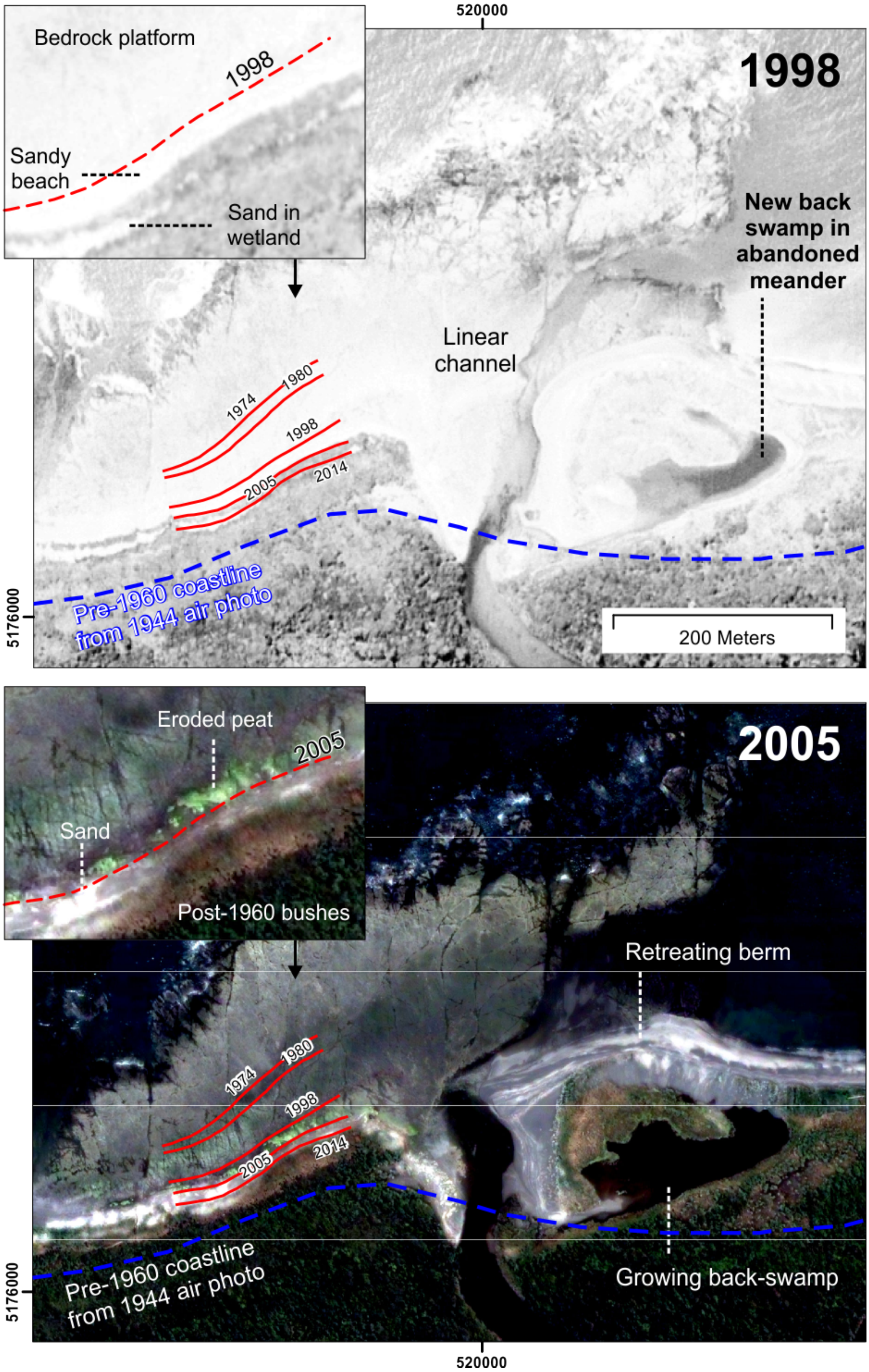

Supplementary Figure 7b. Vertical air photos from 1998 and 2005 (©2008 SAF, ©2006 DigitalGlobe, Supplementary Table 1) showing the main geomorphic features and interpreted beach-bedrock platform limits used to estimate relative sea-level changes from historical air photos. These images include material copyright of SAF and DigitalGlobe, Inc., All Rights Reserved, used with permission under a NERC-BAS educational license and not included in the Creative Commons license for the article.

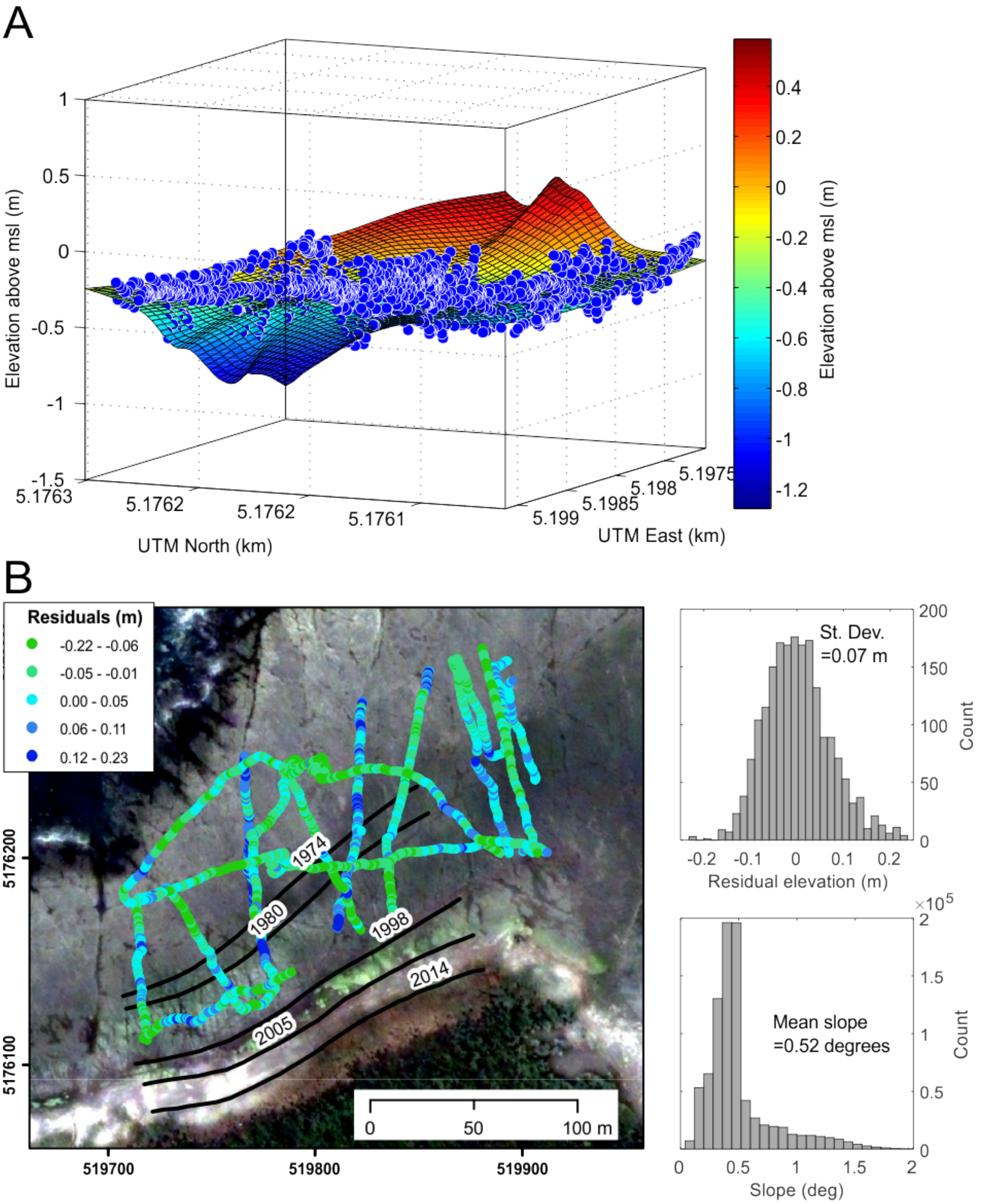

Supplementary Figure 8. Topographic data surveyed with a differential GPS used for surface fitting using local linear regression. A: 3D view showing surface and data. B: Quickbird image (©2006 DigitalGlobe) with GPS data color-coded by the residual value from surface fitting. Note that GPS points were exclusively surveyed on the bedrock platform. Inset shows histogram of residuals. Red curve shows theoretical normal distribution. The fact that residuals are not significantly different from a normal distribution emphasizes the goodness of fit. StDev-standard deviation. This image includes material copyright of DigitalGlobe, Inc., All Rights Reserved, used with permission under a NERC-BAS educational license and not included in the Creative Commons license for the article.

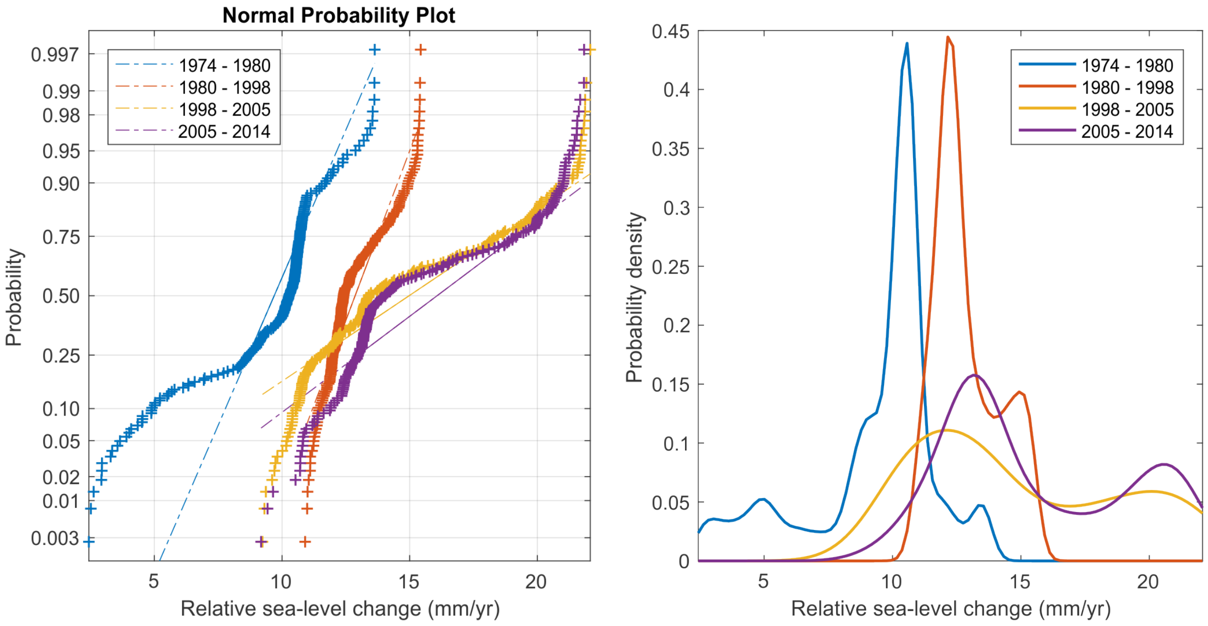

Supplementary Figure 9. Probability plots of relative sea-level change rates from distributions shown in Fig. 2b. Note the increase in relative sea-level change with time for each pair.

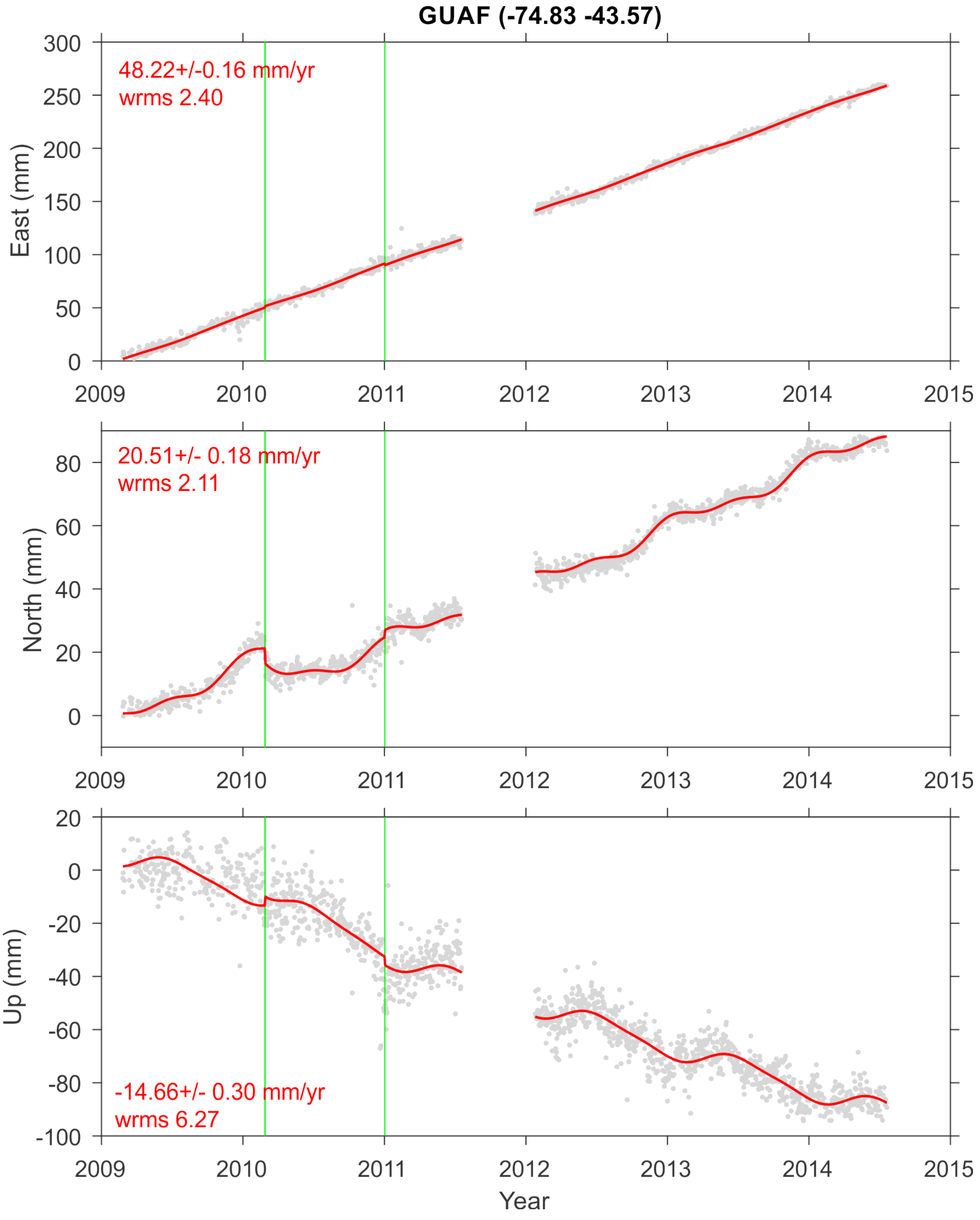

Supplementary Figure 10a. Daily position time series of continuous GPS station GUAF located at the Isla Guafo Lighthouse, hosted by the Chilean Navy. Red line shows trajectory model computed using the ELTM Model[2]. For details see Ref. [3]. Green lines represent nearby earthquakes from the NEIC Catalogue. wrms-weighted root mean square error.

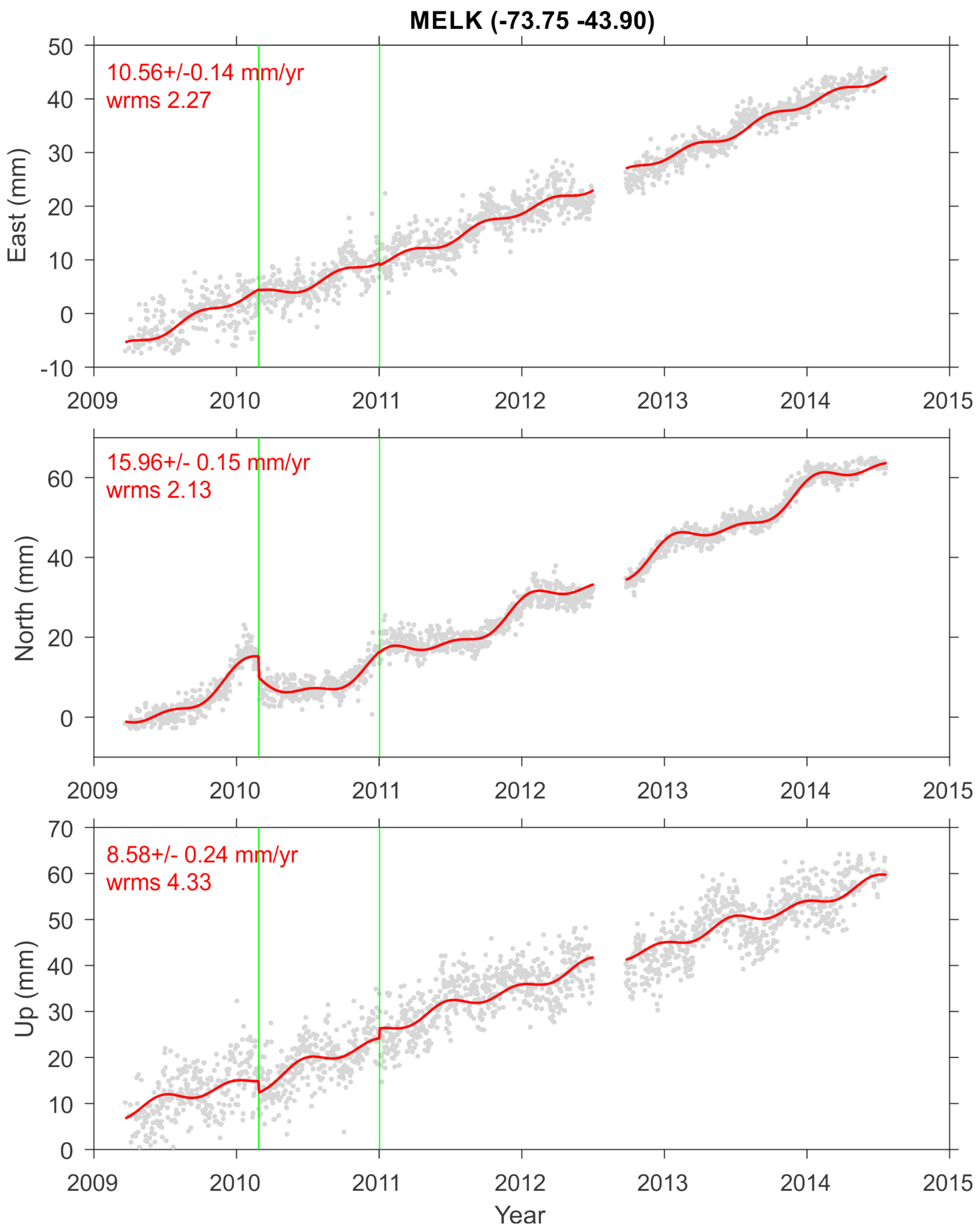

Supplementary Figure 10b. Daily position time series of continuous GPS station MELK located at Melinka Harbor, hosted by the Chilean Navy. Red line shows trajectory model computed using the ELTM Model[2]. For details see Ref. [3]. Green lines represent nearby earthquakes from the NEIC Catalogue. wrms-weighted root mean square error.

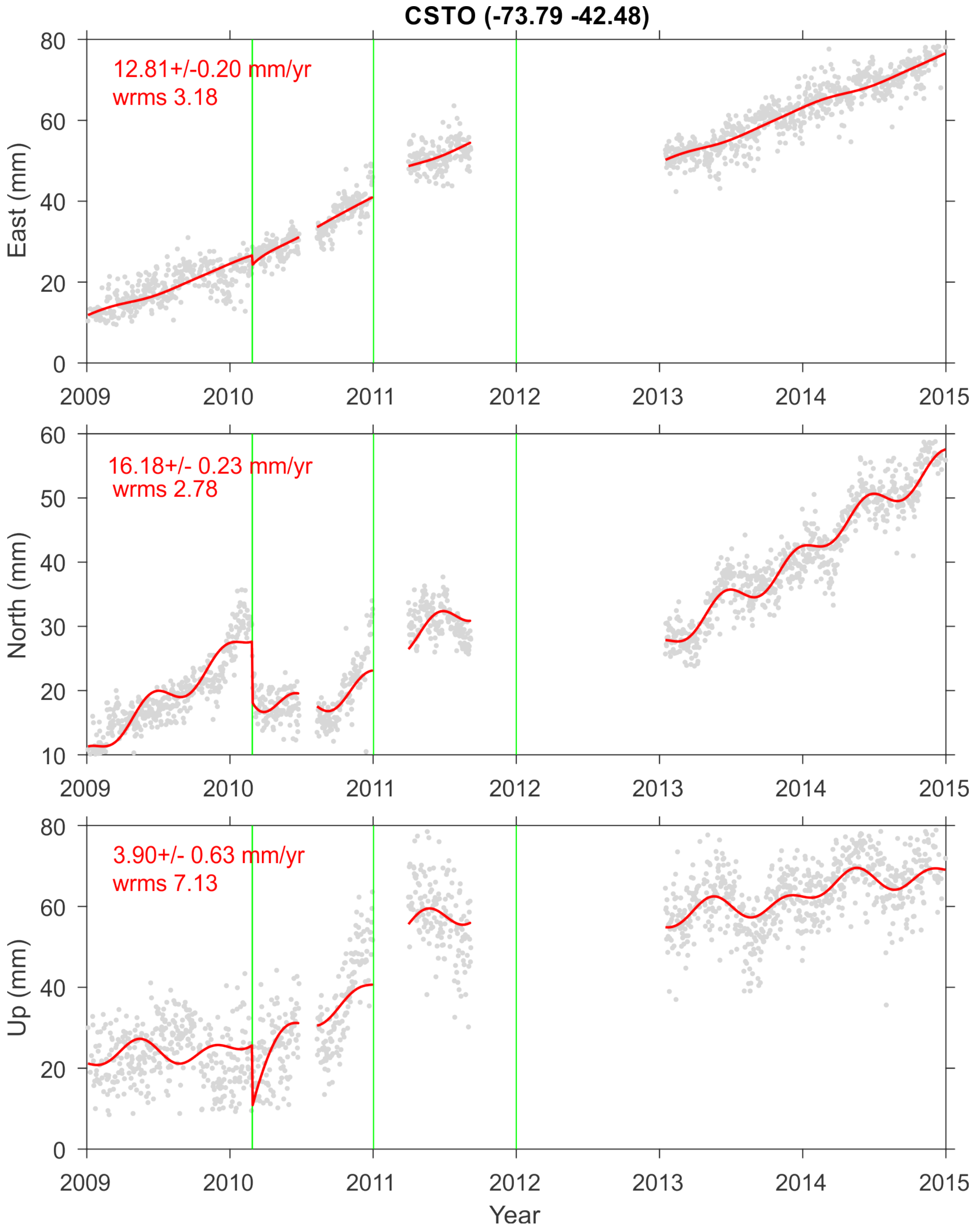

Supplementary Figure 10c. Daily position time series of continuous GPS station CSTO located at Castro. This station is a composite between site CSTR (2009-2012) and BN20 (2013-2015). Red line shows trajectory model computed using the ELTM Model[2]. For details see Ref. [3]. Green lines represent nearby earthquakes from the NEIC Catalogue. wrms-weighted root mean square error.

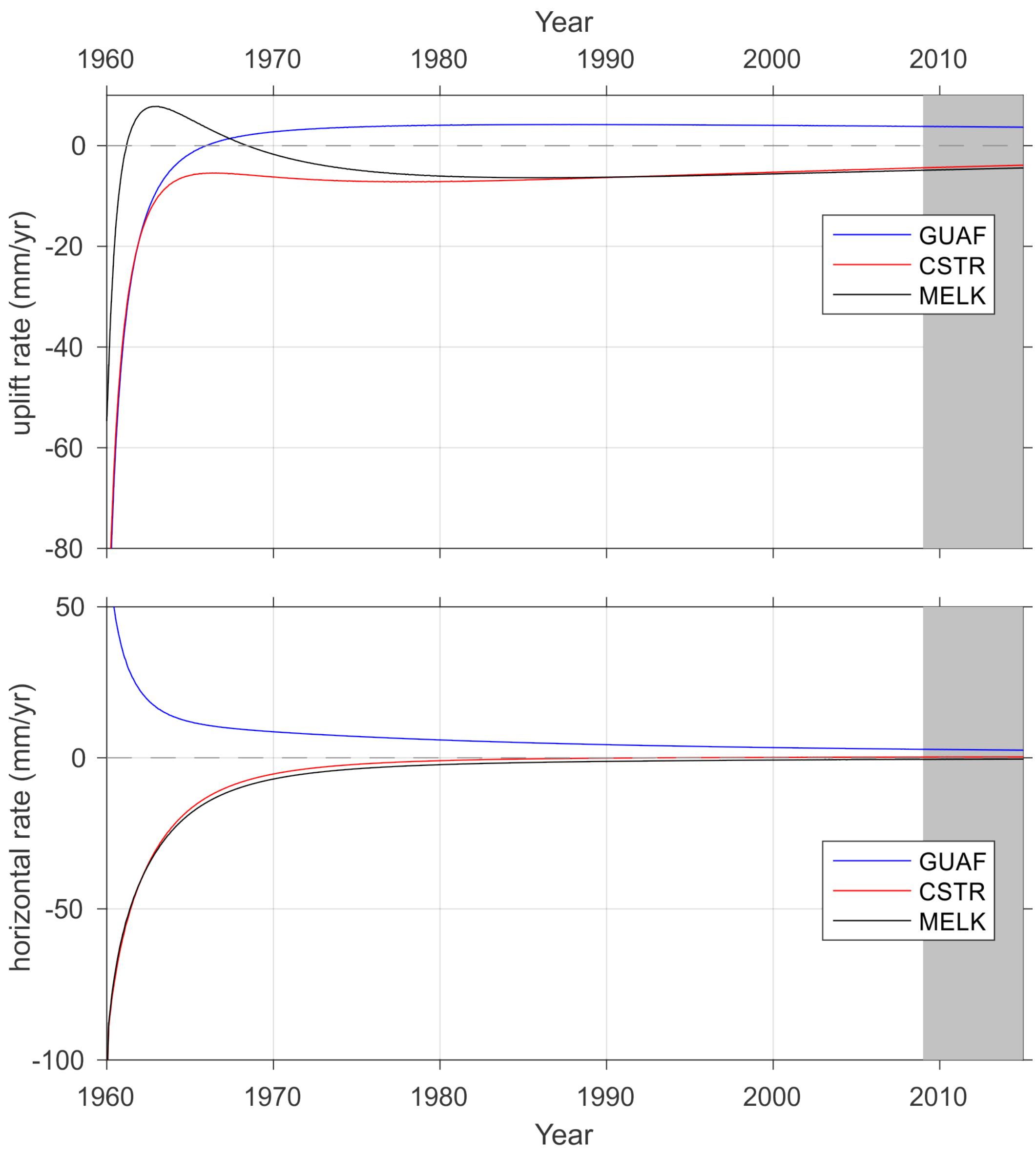

Supplementary Figure 11. Displacement rates estimated by post-seismic model of mantle relaxation at the three continuous GPS stations used in this study. Modeling as in Ref. [4], see Methods for details. Viscosities may be found in Supplementary Table 2. Grey area shows temporal extent of continuous GPS stations in Fig. 4.

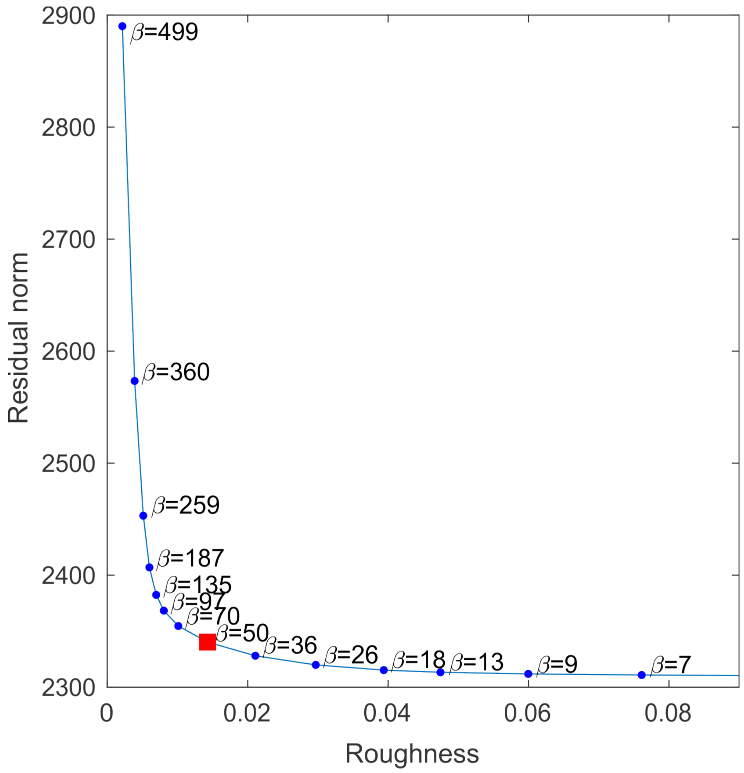

Supplementary Figure 12. Trade-off curve of inverse model for broad range of smoothing parameter (β). The optimal value for β is 50 located at the inflection of the curve denoted by red square.

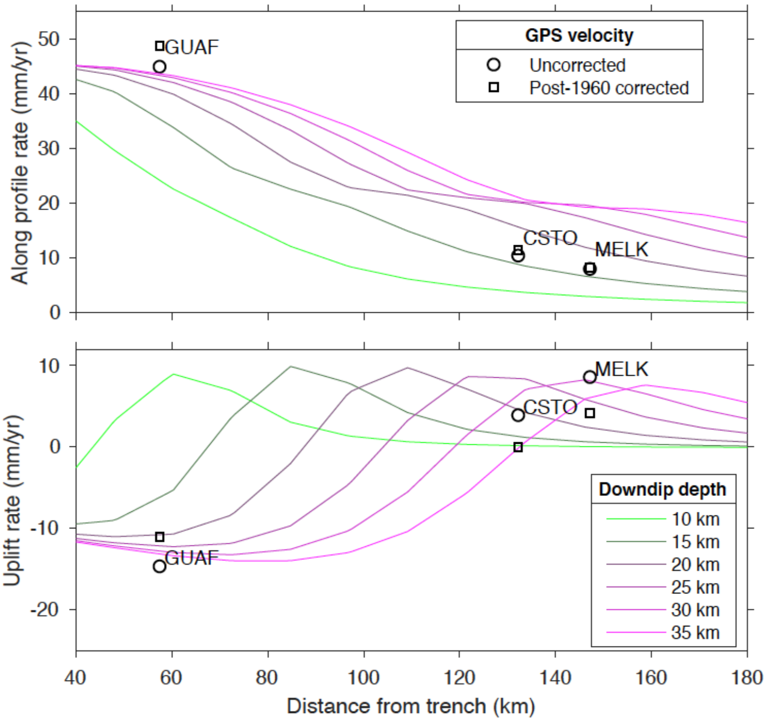

Supplementary Figure 13. Forward model sensitivity to downdip locking depth. All models include a downdip transition zone of 5 km and a fixed updip depth of 5 km.

Supplementary References

1 Farr, T. G. *et al.* The shuttle radar topography mission. *Reviews of geophysics* **45** (2007).

2 Bevis, M. & Brown, A. Trajectory models and reference frames for crustal motion geodesy. *Journal of Geodesy* **88**, 283-311 (2014).

3 Melnick, D. *et al.* The super-interseismic phase of the megathrust earthquake cycle in Chile. *Geophysical Research Letters* **44**, 784-791, doi:10.1002/2016GL071845 (2017).

4 Li, S. *et al.* Postseismic uplift of the Andes following the 2010 Maule earthquake: Implications for mantle rheology. *Geophysical Research Letters* **44**, 1768-1776, doi:10.1002/2016GL071995 (2017).
